# Considerations for diverse, equitable, and inclusive school food programs in the USA and Canada

**DOI:** 10.1093/heapro/daaf015

**Published:** 2025-04-11

**Authors:** Preetama Badyal, Tina Moffat

**Affiliations:** Schulich School of Medicine and Dentistry, Western University, 1151 Richmond Street, London, Ontario, N6A 3K7, Canada; Department of Anthropology, McMaster University, 1280 Main Street West, Hamilton, Ontario, L8S 4L9, Canada

**Keywords:** school food programs, DEI, EDI, nutrition equity, school lunch, school breakfast

## Abstract

School food programs have been shown to support the nutrition of children and their long-term health outcomes in tandem with reducing nutritional inequities experienced by low-income, food insecure, and racialized populations. Understanding the specific needs and outcomes of these equity-deserving groups is crucial when enhancing program implementation and participation in school food programs. A scoping review of equitable, diverse, and inclusive considerations for school food programs was conducted on Canadian and American peer-reviewed and grey literature. The search strategy identified 18 peer-reviewed publications and three grey literature reports that supported the creation of five themes to be explored for school food programs: universal access, food preparation and delivery, sociocultural food preferences, partner involvement, and equitable nutrition. Analysis revealed that while literature surrounding these themes is developing, they serve as a crucial starting point for further research and consideration of the enhancement of school food programs. These themes can support the delivery of a program that is accessible to all students, accommodates their individualized needs, and is free of stigma.

**Contribution to Health Promotion**

- Identifying factors that foster an equitable, diverse, and inclusive school food program can promote the social and health equity of a variety of student populations that may have been previously underrepresented in the literature.
- This review takes a holistic approach to understanding how students, their families, and school staff can be involved and how social determinants of health can be considered in the design of equitable and inclusive school food programs.
- This analysis may be useful for researchers, policymakers, government officials, and school administrators and adapted according to community and school-specific needs.

## INTRODUCTION

An important aspect of school food programs includes their ability to address social disparities in equity-deserving populations. Sources from Canada define an equity-deserving group (also known as an equity-denied or equity-seeking group) as individuals who experience barriers when accessing resources and opportunities that are available to other members or groups of society (Government of Canada 2024). This lack of access is attributed to systemic discrimination and its associated challenges. Equity-deserving groups include individuals with disabilities, women, religious minority groups as well as Indigenous peoples and racialized and/or ethnic minorities. In the context of school food programs, this definition can also include people living in low-income and food-insecure households, particularly given the intersection between the aforementioned groups and socioeconomic status (Cheng et al. 2015, Hernandez et al. 2018).

School food programs have been shown to support children’s nutrition and long-term health outcomes (Hernandez et al. 2018). Nutrient deficiencies or malnutrition early in life can negatively impact aspects of a child’s health, and the school environment serves as an essential place to address this given that students spend most of their waking hours there (Kristjansson et al. 2007, Hernandez et al. 2018). An individual’s health can also be disproportionately affected by factors such as their socioeconomic position. Intergenerational influences experienced by racialized and ethnic minorities may predispose a population subgroup to negative health outcomes or lower social positions, thereby lowering the quality of life for many members of these groups (Cheng et al. 2015, Colley et al. 2019).

School food initiatives in the USA to support nutritional health and address social inequities include the National School Lunch Program (NSLP) and the School Breakfast Program (SBP), both administered by the United States Department of Agriculture, (2017, 2024b). The NSLP was first established in 1946 and the SBP began in 1966 as a pilot project that continues today. Both programs provide low-cost or free meals to students in public and non-profit schools based on income criteria and are implemented in conjunction with federal nutrition requirements and menu decisions made by local school food authorities. In 2014, the Community Eligibility Provision (CEP) was established to support the NSLP and SBP, depending on eligibility criteria at the level of the student(s) and school to offer free breakfast and lunch meals to all students (United States Department of Agriculture, 2023, 2024a). This initiative streamlines administrative work required for meal programs (e.g. less paperwork for determining eligibility) and minimizes stigma associated with the tiered system (i.e. free versus reduced-price meals) in pre-existing food programs.

Canada is the only G7 country without a government-funded school food program (Zhong et al. 2023). On 1st April 2024, the Canadian federal government announced a plan to invest 1 billion dollars over the next 5 years towards developing a national school food program in partnership with provinces, territories, and Indigenous partners (Office of the Prime Minister of Canada 2024). A National School Food Policy highlighting principles for implementation was published in June 2024 by the Government of Canada (Employment and Social Development Canada 2024). As of 2023–24, only 52% of students in Canada had access to school food programs (Ruetz Consulting, 2024). These programs are funded partly by government as well as community groups, charities, and private sector organizations, and rely on the support of volunteers (Haines and Ruetz 2020, Ruetz and McKenna 2021). There are also inconsistencies in program standards and delivery, causing disparities in how the social, cultural, and nutritional needs of Canadian children are met (Haines and Ruetz 2020).

Despite policy and program differences, the USA and Canada face similar challenges in their respective school food environments, particularly in terms of social and nutritional inequalities among school-aged children (Zhong et al. 2023, Ruetz and Poppendieck 2024). Therefore, there is an immense opportunity to learn from research on programs in both countries. Additionally, while a common recommendation for school food programs globally has been to implement universal access to meals, the incorporation of considerations for a more diverse, equitable, and inclusive policy against the backdrop of existing traditional frameworks challenges the current one-size-fits-all model. A review of current research in school food programs that considers the lived experiences, needs, and preferences of equity-deserving groups and their affiliated communities in the USA and Canada can inform best practices for program design and evaluation. This scoping review aims to explore facilitators and barriers that could influence the implementation of diverse, equitable, and inclusive (DEI, also known as EDI) school food programs for children in elementary, middle, and high schools. Secondary objectives include assessing the current state of literature in the USA and Canada surrounding these considerations, as well as determining the central perspectives from parents/caregivers, school staff, and school-aged children concerning school food program implementation.

## METHODS

Arksey and O’Malley’s Methodological Framework for Scoping Reviews alongside the PRISMA-ScR (Preferred Reporting Items for Systematic reviews and Meta-Analyses extension for Scoping Reviews) checklist were used to inform the search protocol (Arksey and O’Malley 2005, Tricco et al. 2018). The search included peer-reviewed and grey literature published in English from January 2000 to March 2024. Given the authors’ understanding of developments in the literature surrounding DEI principles alongside changing policy regarding school food, a search for literature within the 21st century was selected (Hirschman and Chriqui 2012, Tricco et al. 2018). Included literature discussed school food programs in the USA or Canada and included an outcome assessment or discussion concerning the program participation of equity-deserving groups and diverse populations. Sources examining elementary and/or middle school children (kindergarten to grade 8) alongside high school student populations (grades 9–12) were included for review. Sources were excluded if they did not consist of primary research (e.g. literature reviews), solely reported outcomes on high school populations, or discussed the school food environment without mention of a school food program.

The databases searched included those located on EBSCOhost Databases, CINAHL (separate search to consider subject headings), Web of Science, Scholars Portal Journals, Scholars Portal Books, Ovid MEDLINE (1946–present), JSTOR, and ERIC. The search strategy (Table 1) was developed with the assistance of a university librarian team and adapted for each database. Date and language parameters were applied to meet the inclusion criteria. The peer-reviewed search was conducted between 22 February 2024 and 24 February 2024. The grey literature search, conducted in March 2024, was adapted from the peer-reviewed search strategy and considered national and provincial/state organizations that met the geographical inclusion criteria. These resources (highlighted in Table 2) were informed and reviewed by the second author (T.M.) and academic librarians. A search was conducted in Google Scholar to find any sources missed in the previously stated searches (Arksey and O’Malley 2005). This was informed by best practices in the literature for systematic searches which included a planned title search beyond the first 200–300 results (Haddaway et al. 2015). Although these guidelines were established ahead of the search, less than 200 results were found so a definitive limit was not set, and the entire search yield was reviewed. Finally, the references of included literature were reviewed to identify additional sources.

**Table 1.** Ovid MEDLINE search strategy.

exp Canada/ Canad*.mp. exp Alberta/ Albert*.mp. exp British Columbia/ British Columbia*.mp. exp Manitoba/ Manitob*.mp. exp New Brunswick/ New Brunswick.mp. exp Newfoundland and Labrador/ Newfoundland*.mp. exp Northwest Territories/ Northwest Territories.mp. exp Nova Scotia/ Nova Scotia.mp. exp Nunavut/ Nunavut.mp. exp Ontario/ Ontari*.mp. exp Prince Edward Island/ Prince Edward Island.mp. exp Quebec/ Quebec.mp. exp Saskatchewan/ Saskatchewan.mp. exp Yukon Territory/ Yukon Territory.mp. USA.mp. exp United States/ United States*.mp. Americ*.mp. 1 or 2 or 3 or 4 or 5 or 6 or 7 or 8 or 9 or 10 or 11 or 12 or 13 or 14 or 15 or 16 or 17 or 18 or 19 or 20 or 21 or 22 or 23 or 24 or 25 or 26 or 27 or 28 or 29 or 30 or 31 or 32 Schools/ elementary school.mp. primary school.mp. Students/ grade school.mp. exp Child/ middle school.mp. K-8.mp. 34 or 35 or 36 or 37 or 38 or 39 or 40 or 41 exp Meals/ school meal.mp. exp Nutrition Policy/ school food.mp. meal program.mp. nutrition program.mp. food program.mp.
snack program.mp. breakfast program.mp. lunch program.mp. school lunch.mp. 43 or 44 or 45 or 46 or 47 or 48 or 49 or 50 or 51 or 52 or 53 exp Social Stigma/ stigma*.mp. exp Prejudice/ prejudice.mp. discriminat*.mp. exp Culture/ cultur*.mp. divers*.mp. exp cultural diversity/ exp diversity, equity, inclusion/ inclusiv*.mp. equit*.mp. exp Poverty/ poverty.mp. low-income.mp. SES.mp. exp low socioeconomic status/ exp Socioeconomic Factors/ exp socioeconomic disparities in health/ socioeconomic*.mp. socioeconomic status.mp. exp Ethnic and Racial Minorities/ exp Racial Groups/ racial*.mp. race.mp. exp Ethnicity/ ethnic*.mp. EDI.mp. DEI.mp. access*.mp. 55 or 56 or 57 or 58 or 59 or 60 or 61 or 62 or 63 or 64 or 65 or 66 or 67 or 68 or 69 or 70 or 71 or 72 or 73 or 74 or 75 or 76 or 77 or 78 or 79 or 80 or 81 or 84 33 and 42 and 54 and 85 limit 86 to (english language and yr=”2000 - 2024”)

**Table 2.** Grey literature search strategy.

USA organizations (Web search)	Canadian organizations (Web search)	Other
FoodCorps	Health Canada	Google Scholar (Advanced Search)
USDA Food and Nutrition Service	Food Secure Canada (Including the Coalition for Healthy School Food)	
School Nutrition Association	Centre for Family Equity	
Food Research and Action Center		

**Figure 1.**
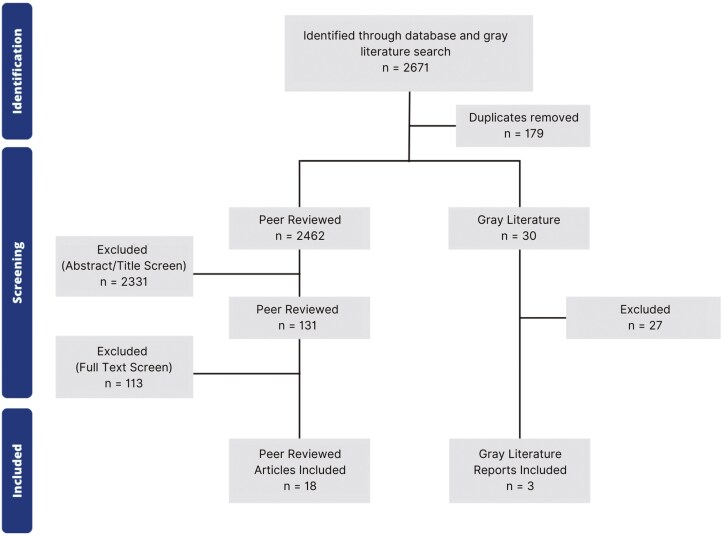
Flowchart of study selection.

Screening consisted of a title, abstract, and full-text evaluation conducted through Covidence by one reviewer (P.B.). Conflicts as well as articles denoted as ‘maybe’ were addressed by the authors together (P.B., T.M.). Duplicates were removed by the Covidence software (Fig. 1). A data chart was developed using Google Sheets to extract selected articles and data charting was done independently by one author (P.B.). The variables in this process included the article title, author(s), year of publication, country/region of study, purpose, population and sample size, methodology, outcome measurements, and main findings that addressed the review objectives.

A critical appraisal of the included literature was not conducted given the conventional methodology of a scoping review (Tricco et al. 2018). To synthesize the results of the search, thematic analysis was manually adapted by one author (P.B.) and reviewed by both authors (P.B., T.M.). This methodology was informed by the theoretical framework provided by Braun and Clarke (2006; Clarke and Braun, 2017). Analysis was completed by hand (i.e. through documentation and manual categorization) and involved initially taking detailed notes on literature results (including descriptive statistics, quantified trends, and survey or interview responses). Given the size of the literature yield, manual analysis was feasible as well as allowed for broader interpretations given the intent of the review to explore DEI considerations that may be more nuanced in the observed data. To account for the variety of research strategies included in this review (i.e. qualitative, quantitative, and mixed methods), themes were generated from numerical data in the form of observed trends, whereas narratives were analyzed for keywords and associated patterns to ensure the application of the framework was as consistent as possible across studies. The authors (P.B., T.M.) discussed and refined the final list of thematic results together by independently comparing generated themes against the results of each study, and meeting to validate findings as well as resolve any discordant interpretations.

## RESULTS

### Summary of included literature

The search strategy and screening process resulted in the retrieval of 21 primary data publications, including 18 peer-reviewed articles and three grey literature reports (Fig. 1). Table 3 summarizes the individual characteristics of each source, including its publication type (peer-reviewed or grey literature), year of publication, country/region, objectives, discussed demographics, program type(s) mentioned, and allocated theme(s). The included literature was published from August 2011 to March 2024, and included quantitative (*n* = 12), qualitative (*n* = 4), and mixed-methods research (*n* = 5). School food programs in this review included breakfast, lunch, and snack programs, with some studies including more than one type (e.g. breakfast and lunch).

**Table 3.** Summary of included articles.

Peer-reviewed (PR) or grey literature (GL)	Reference; country, region	Objectives	Demographics discussed	Program type(s)	Identified themes
PR	(Andreyeva and Sun 2021); USA, National	Assessment of the Community Eligibility Provision on school meal participation, educational outcomes, body weight, and household food security.	Elementary school students (race/ethnicity, language at home, income, food insecurity, disability, urbanicity)	Lunch	Universal access
	(O’Neill et al. 2020); USA, California	Assessment of the ‘Rethinking School Lunch Oakland’ (RSLO) approach to providing food to students and families ahead of a program infrastructure project.	Elementary and middle school students, parents, and school personnel (race/ethnicity, food insecurity, language at home)	Lunch	Food preparation and delivery
	(Spruance and Vo 2022); USA, National	Assessment of the association between school meal participation and acculturation, race, and ethnicity.	Middle school students (race/ethnicity, acculturation variables)	Breakfast, lunch	Sociocultural food preferences
	(Adams et al. 2021); USA, Virginia	Examine and compare nutrients in school lunch preferences/consumption to recommendations.	Elementary school students (race/ethnicity)	Lunch	Equitable nutrition
	(Jowell et al. 2021); USA, California	Examine facilitators and barriers to providing school meals during the COVID-19 pandemic.	Elementary, middle, and high school students. Data collected from parents and school district personnel (race/ethnicity, language at home, food insecurity, urbanicity)	Meal (primarily lunch), snack	Food Preparation and Delivery; Partner Involvement; Sociocultural Food Preferences
	(Bailey-Davis et al. 2013); USA, Pennsylvania	Explore the perceptions of low-income, urban parents and youth regarding school breakfast programs.	Elementary and middle school students, as well as parents (race/ethnicity, income)	Breakfast	Universal access; food preparation and delivery; sociocultural food preferences
	(Turner et al. 2016); USA, National	Examine changes in lunch to see persisting disparities by school/student demographics after changed USDA standards in 2013.	Elementary school students (race/ethnicity, urbanicity, socioeconomic status)	Lunch	Equitable nutrition
	(Peckham et al. 2019); USA, South Carolina	Assess selection and consumption behaviours of National School Lunch Program (NSLP) participants according to demographic data.	Elementary school students (race/ethnicity, eligibility for program)	Lunch	Equitable nutrition
	(Askelson et al. 2017); USA, Midwest	Examine perceptions, attitudes, and beliefs related to the School Breakfast Program (SBP) held by school administrators.	Elementary, middle, and high school students. Data obtained from school administrators (urbanicity)	Breakfast	Universal access; food preparation and delivery; sociocultural food preferences; partner involvement
	(Bhatia et al. 2011); USA, California	Evaluation of stigmatization of NSLP participation after removal of competitive lunch options.	Middle and high school students (eligibility for program).	Lunch	Food preparation and delivery
	(Chandrasekhar et al. 2023); USA, Texas	Evaluation of the impact of the Breakfast After The Bell (BATB) program on educational outcomes.	Elementary, middle, and high school students (race/ethnicity, socioeconomic status/eligibility for program)	Breakfast	Food preparation and delivery
	(Cohen et al. 2022); USA, Maine	Evaluation of the impact of implementing universal school meals (USM) amidst and beyond the COVID-19 pandemic.	Elementary, middle, and high school students. Data collected from school food authorities (SFAs) (race/ethnicity, urbanicity)	Breakfast, lunch	Universal access
	(Gearan et al. 2020); USA, National	Assessment of NSLP participation and diet quality of students depending on demographic factors.	Elementary, middle, and high school students (race/ethnicity, income, eligibility for program)	Lunch	Equitable nutrition
	(Ohri-Vachaspati 2014); USA, New Jersey	Assessment of parental perception of nutritional quality in school meals and student participation in the lunch program.	Elementary, middle, and high school students (income, race/ethnicity, program eligibility, parental background)	Lunch	Partner involvement
	(Ismail et al. 2023); Canada, Ontario	Examined student opinions and recommendations for the Ontario Student Nutrition Program (OSNP).	Elementary school and middle school students (urbanicity)	Meal (not specified; typically breakfast), snack	Food preparation and delivery; partner involvement
	(McIsaac et al. 2017); Canada, Nova Scotia	Aimed to explore how school food programs in Nova Scotia may contribute to stigmatization and social exclusion.	Elementary, middle, and high school students. Data collected from parents, school personnel, and volunteers (urbanicity)	Breakfast, snack, lunch	Universal access; partner involvement
	(Ismail et al. 2021); Canada, Ontario	Process evaluation of the Centrally Procured School Food Program (CPSFP).	Elementary and middle school students. Data collected from parents, school personnel, and volunteers (urbanicity)	Snack	Universal access; food preparation and delivery; partner involvement
	(Datta Gupta et al. 2023); Canada, Saskatchewan	Examine factors influencing participation and willingness to pay (WTP) for a cost-shared school food program.	Elementary and middle school students. Data collected from parents/caregivers (income, race/ethnicity, food insecurity, neighbourhood income)	Meal (not specified; typically lunch)	Universal access; partner involvement
GL	(Alemayehu 2022); Canada, British Columbia	Highlight the perceptions and experiences of low-income caregivers in universal school food.	Elementary, middle, and high school students. Data obtained from parents and caregivers (race/ethnicity, income, disability, single parent)	Breakfast, lunch, snack	Universal access; food preparation and delivery; sociocultural food preferences; partner involvement
	(Employment and Social Development Canada 2023); Canada, National	Summarize experiences and perceptions of developing a National School Food Policy and program.	Elementary, middle, and high school students. Data gathered from various partners (representation from various levels of urbanicity, race/ethnicity, income levels)	Breakfast, lunch, snack	Universal access; food preparation and delivery; sociocultural food preferences; partner involvement; equitable nutrition
	(Food Research & Action Center 2024); USA, National	Measure participation of breakfast and lunch programs in the 2022–23 school year following the COVID-19 pandemic.	Elementary, middle, and high school students (varied demographic data, discusses income)	Breakfast, lunch	Universal access; food preparation and delivery

The peer-reviewed literature included 14 studies located in the USA, with four highlighting nationally representative samples (categorized as ‘National’), three from California, and one each from New Jersey, Maine, Texas, South Carolina, Pennsylvania, Virginia, and an unspecified Midwestern state. Four studies from Canada were retrieved, including two from Ontario, and one each from Nova Scotia and Saskatchewan. Methods used in these studies included data collection from local, state, and federal databases (primarily school, student, and program-level information), interviews, mealtime observations, dietary recalls, surveys (online and phone), focus groups, and the assessment of digital images of meals (Bhatia et al. 2011, Bailey-Davis et al. 2013, Ohri-Vachaspati 2014, Turner et al. 2016, Askelson et al. 2017, McIsaac et al. 2017, Peckham et al. 2019, Gearan et al. 2020, O’Neill et al. 2020, Adams et al. 2021, Andreyeva and Sun 2021, Ismail et al. 2021, 2023, Jowell et al. 2021, Cohen et al. 2022, Spruance and Vo 2022, Chandrasekhar et al. 2023, Datta Gupta et al. 2023).

The grey literature included one national report from the USA and two reports from Canada, one with national data and one from British Columbia. Methods used in these reports included data collection from local, provincial/state, and federal databases, partner roundtables and meetings, visits to schools, a public questionnaire, written submissions, focus groups, and advisory councils (Alemayehu 2022, Employment and Social Development Canada 2023, Food Research & Action Center 2024).

The peer-reviewed literature dataset included findings from elementary, middle, and high school students (many including two or more of these education levels) and demographics such as ethnicity/race (primarily reported in the included studies as non-Hispanic Black, non-Hispanic White, and Hispanic but also Asian, Latino/Latin American, multiracial, and Indigenous populations), language spoken at home, income, food insecurity, individuals with disabilities (child or caregiver), urbanicity, acculturation variables, socioeconomic status, parental background (including single-parent families), and regional/neighbourhood income. (To fully reflect the findings from the literature, we employ the terminology associated with ethnic/racial/sociodemographic categories and groups used in the included studies in this review article.) Perceptions and opinions reported in these publications included those of students, parents/caregivers, school staff, program volunteers, school food authorities, community organizations (health, food and agricultural, service delivery, charities, etc.), experts from advisory councils, academics, government representatives, and Indigenous community partners.

**Figure 2.**
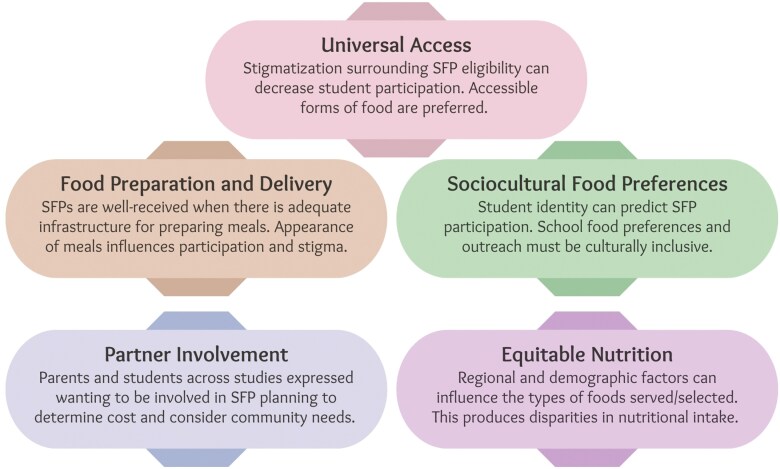
Considerations for an equitable, diverse, and inclusive school food program.

Five prominent themes were identified through data analysis: universal access, food preparation and delivery, sociocultural food preferences, partner involvement, and equitable nutrition. These themes are elaborated on below and summarized in Fig. 2.

### Universal access

Eight peer-reviewed articles and all three grey literature reports (*n* = 11 publications) discussed the importance of universally accessible school meals, i.e. school meals that are available to all children regardless of income or sociodemographic position. Despite the widespread availability of American programs such as the SBP and NSLP, several studies demonstrated that some students who are eligible for free or reduced-price meals do not participate due to concerns related to perceived or experienced stigma among school peers (Bailey-Davis et al. 2013, Askelson et al. 2017, Cohen et al. 2022). An intervention program found that the removal of the à la carte lunch system increased universal accessibility, highlighting how the à la carte lunch system (i.e. competitive food offerings that can be bought alongside free meals) has the potential to propagate stigma and discourage participation in school food programs. Since competitive foods are not as accessible to low-income students, due to the associated cost of à la carte offerings, students who are receiving free or subsidized lunches without buying à la carte items become more identifiable, therefore, introducing the potential for stigmatization (Bhatia et al. 2011). In one study, school administrators also perceived that social messaging surrounding school breakfast hindered participation, particularly assumptions that access to these programs is a marker of poverty (Askelson et al. 2017). This is notable because participation in the SBP is lower than the NSLP; less stigmatized programs as well as alternate forms of access such as ‘grab-and-go’ breakfasts can potentially mitigate this (Bailey-Davis et al. 2013, Food Research & Action Center 2024).

Universal access can also be impacted by the tiered payment system that exists in many programs in the USA. Through the allocation of means-tested benefits, families at risk of food insecurity or those that could benefit from additional resources such as meal programs may still struggle to afford participation costs if they fall just above income thresholds, ultimately leading to exclusion (Cohen et al. 2022). Universal free meals were provided in the USA to high-poverty schools from 2014 onwards through the Community Eligibility Provision (CEP). One study measured the effects of the CEP on equity-deserving groups and found increased school meal participation and a modest improvement in school attendance (Andreyeva and Sun 2021). Participation in the CEP, moreover, had a statistically significant effect on reducing the probability of being overweight in low-income families and Hispanic children also displayed a marginally significant increase in their reading scores.

The discussion surrounding universally accessible school meals was prevalent during the COVID-19 pandemic with the availability of CEP to all schools; many schools opted for free school meals due to waivers put in place by the USDA (Cohen et al. 2022, Food Research & Action Center 2024). A study by Cohen et al. (2022) reported perceptions from school food authorities following changes in program delivery during the pandemic, including an increase in participation and perceived reduction in stigma. This is notable as the pandemic caused a rise in household food insecurity, which disproportionately impacted communities of colour. These challenges continue to persist or may have even been exacerbated with the expiration of these waivers (Food Research & Action Center 2024). Following the return to normal operations in the 2022–23 school year, SBP and NSLP participation decreased by 1.2 million (7.7%) and 1.8 million children (6.0%), respectively. Some states, however, increased CEP participation following the pandemic (e.g. California, Maine, Massachusetts, Vermont, and Pennsylvania) and implemented a Healthy School Meals for All/Healthy School Breakfast for All policy to continue offering free meals to all students in a significant proportion of their schools (Food Research & Action Center 2024).

Many of the Canadian school food programs assessed in the peer-reviewed literature provided universal access, even if it was through an informal community-based program (McIsaac et al. 2017, Ismail et al. 2021). Some studies, however, highlighted how these programs can contribute to social division and stigmatization for those experiencing food insecurity (McIsaac et al. 2017, Ismail et al. 2021, Alemayehu 2022, Employment and Social Development Canada 2023). A study by McIsaac et al. (2017) commented on how suggestions from parents and volunteers to promote educational solutions for poor nutrition ignored systemic factors related to food insecurity. It was also found in this study that a student’s food security status could be ‘invisible’ due to sacrifices from other areas of their household or families simply not asking for support, a barrier that could be further removed through universal access (McIsaac et al. 2017). Other reported stigmatizing barriers included a lack of communication surrounding available subsidies and confusion and humiliation (Alemayehu 2022). Program personnel can also serve as a barrier to providing universal access due to skewed expectations about the program (e.g. thinking food programs are only for poor students) (Ismail et al. 2021, Employment and Social Development Canada 2023). Suggestions from surveys of the Canadian public to implement a universally accessible program without stigmatization included prioritizing public funding for students within a certain grade, school, or neighbourhood, implementing a ‘pay what you can’ model, and providing the same meal options and treatment for all students (Employment and Social Development Canada 2023). Payment models and parents’ willingness to pay for a cost-shared school food program were explored by Datta Gupta et al. (2023). In their survey, they found that having a higher income, being food secure, and having multiple children predicted a willingness to pay more for school food programs. The authors stated that a cost-shared program would be more sustainable if community characteristics were kept in mind when determining price. This was consistent in other literature, where parent perspectives revealed that paying for current school meal programs is out of reach for low-income families (Alemayehu 2022). Reported perspectives from children showed agreement with universal access and the belief that there should be free options for those who cannot pay. Some children expressed guilt about participating and wanted to leave food for others who may not have access at home, indicating the need for more food to be made available for all students (Employment and Social Development Canada 2023).

### Food preparation and delivery

It is also important that school food programs are delivered in a way that encourages participation and decreases stigmatization. This can be done through innovative meal models, providing students with enough time to eat, and providing appealing food. Eight peer-reviewed articles and all three grey literature reports (*n* = 11 publications) highlighted the benefits of equitable food preparation and delivery. In the USA, it was found that the presentation and preparation of school food have an impact on its perception and consumption by students (O’Neill et al. 2020). A lack of cooking kitchens at high-poverty sites alongside prepackaged food was shown to be correlated with low student consumption and negative responses to school food, resulting in perceived stigma, student teasing, and the characterization of students who eat prepackaged food as ‘desperate’ (Bailey-Davis et al. 2013, Askelson et al. 2017, O’Neill et al. 2020). Parents were concerned about the low nutritional quality and lack of appeal for their children when food was offered as prepackaged or not made on-site (Bailey-Davis et al. 2013, O’Neill et al. 2020). This effect was particularly of note at schools that also sold competitive food offerings, where the variety and delivery of food differed from what was offered by the program (e.g. through the form of payment, portion size, or the presence of a separate line) (Bhatia et al. 2011). Bhatia et al. (2011) showed that students eligible for the NSLP had higher participation rates once competitive lunch choices were removed alongside efforts to have more variety in school program meals. Barriers to program delivery in other locations included a lack of time and resources for staff such as space and funding (Askelson et al. 2017). These were highlighted in rural populations, where students face logistical barriers such as lengthy commutes, limiting the time available for participation in breakfast programs. During the COVID-19 pandemic, many school food programs were more flexible in how meals were distributed; families benefited from adaptable meal schedules and being able to pick up food in batches without their children present (Jowell et al. 2021).

The impact of non-traditional models of access such as providing meals in the classroom, grab and go, and second-chance meals were also studied (Askelson et al. 2017). Chandrasekhar et al. (2023) assessed the impact of the ‘Breakfast After the Bell’ (BATB) program, which gives students another chance to consume breakfast and reduces the stigma associated with programs provided before the beginning of the school day. In a population of racialized and ethnic minorities as well as low-income students, it was found that Asian, non-Hispanic Black, Hispanic, and Indigenous populations were more likely to participate in the BATB program after adjusting for sex and age. These alternative forms of delivery are more effective in providing universal, equitable, and inclusive access to school food programs (Food Research & Action Center 2024).

In the Canadian literature, children expressed a need for increased variety and choice in food offerings alongside appealing and convenient meal presentations (Alemayehu 2022, Ismail et al. 2023). Children wanted foods that were served in a ‘fun’ way, at an affordable price, and in a fresh condition (Employment and Social Development Canada 2023). Parents and school personnel shared similar sentiments but also expressed challenges in procuring physical resources such as food storage infrastructure in schools (Ismail et al. 2021, Employment and Social Development Canada 2023). This was noted as an issue in rural, remote, and Northern communities, where there are higher costs for food, varying levels of infrastructure, and more severe constraints due to harsh winter climates (Employment and Social Development Canada 2023). Inequalities may also exist in the provision of school food programs and perceived meal quality, creating a tiered system where schools or communities might contract to non-profits or for-profits depending on the affluence of the school and thus availability of funding. Caregiver discussions about existing school food programs in British Columbia uncovered concerns that non-profits might provide lower-quality food, though others believed that for-profit contractors might serve lower-quality food to save money (Alemayehu 2022). In those same discussions, some caregivers pointed out that adaptive delivery methods should be made for students who may not always make it to school, such as children with chronic illnesses or disabilities.

### Sociocultural food preferences

Six peer-reviewed articles and two grey literature reports (*n* = 8 publications) noted the importance of accounting for social and cultural preferences at the level of individual student identities as well as school, region, and community-based values. In the USA, there were various outcomes reported on specific racialized and ethnic groups in terms of their perceptions and participation in school food programs. For example, a study based on a survey of middle school students in California found that Southeast Asian, East Asian, Hispanic, South Asian, and mixed-race individuals were less likely to participate in school breakfast, and East and South Asian students were less likely to participate in school lunch (Spruance and Vo 2022). The authors did not investigate the reasons for these disparities in participation. It was also noted that individuals who have spent more time outside of the USA may have a wider palate of preferences and their participation based on taste and appeal may not be the same as other students. The authors argue that differences in participation rates by cultural identity exist, and therefore school food programs should consider interventions to provide equitable opportunities for engagement, such as aligning with cultural preferences. A community-based study from San Joaquin Valley, California noted the importance of multilingual communication (e.g. English and Spanish across diverse platforms) to ensure low-barrier communication and outreach that is culturally as well as linguistically accessible (Jowell et al. 2021). In another study, students and parents presented concerns that preferences would not be met with school food (whether due to cultural acceptance or personal taste) and instead opted to eat at home (Bailey-Davis et al. 2013). One study from Maine reported little to no feedback on meeting students’ cultural preferences in food programs, but this may be because the survey only went out to school food program administrators and the school(s) included in the study had primarily white student populations (Cohen et al. 2022).

Geographical location also influences sociocultural food preferences. Partners in urban regions reported more challenges with accommodating cultural food preferences (Cohen et al. 2022). In rural communities, reported family norms of food provision at home and sharing meals together or community perceptions of self-sufficiency were considered a barrier to participation in school food programs, despite the fact that food insecurity is prevalent in rural populations (Askelson et al. 2017). Understanding these regional characteristics can shape community-specific interventions for school food programs to increase participation and inclusion.

Canadian literature provided more detail on accommodating cultural preferences and reported on racialized and ethnic groups not represented in American publications. This included perspectives from Indigenous populations (Alemayehu 2022, Employment and Social Development Canada 2023). In this literature, there was a focus on Indigenous reconciliation in school food programs, including collaboration with First Nations, Inuit, and Métis community partners to educate children about traditional foods. Parents and other partners such as the school community and advocacy organizations expressed the importance of school food programs to teach children about and represent different cultures and traditions. The importance of social connection through school food programs to lessen cultural division among children was highlighted, alongside the inclusion of families to join children for meals to create community and address household food insecurity (Ismail et al. 2021, Alemayehu 2022, Employment and Social Development Canada 2023).

### Partner involvement

A theme emphasized in eight peer-reviewed articles and two grey literature reports (*n* = 10 publications) was the importance of partnering with those who are currently not actively involved in program development such as parents and children. In the USA, parents reported feeling unheard due to having limited opportunities to provide feedback on school food programs (Jowell et al. 2021). This led to perceptions that school meals do not meet nutritional standards, perpetuating misconceptions and stigmatization surrounding their purpose. A study by Ohri-Vachaspati (2014) found that parental perceptions of the nutritional quality of school meals are positively associated with students’ participation in school food programs, which was interpreted as the need to provide more information about programs to parents. Parents and families, moreover, may not know about the availability of school food programs, which hinders accessibility for equity-deserving groups (Askelson et al. 2017). Previously mentioned concerns about school food preparation can also be mitigated by student involvement in menu planning and communicating preferences to develop socioculturally acceptable options (Bailey-Davis et al. 2013). Therefore, keeping key partners such as parents and students involved in school meal planning is important for inclusive implementation (Bailey-Davis et al. 2013, Ohri-Vachaspati 2014).

Included literature from Canada reported similar outcomes, with food-insecure families having lower engagement in program planning (McIsaac et al. 2017). Several studies reported recommendations to include input from children and parents/caregivers to increase food preferences being met, with a request for more choice in food offerings and effort to meet the needs of children with allergies and cultural preferences (Datta Gupta et al. 2023, Employment and Social Development Canada 2023, Ismail et al. 2023). Parents specifically requested more informal opportunities to communicate the social, cultural, and economic aspects of food for various sociodemographic groups, and to encourage input from families that do not normally engage in these discussions. Conventional methods such as parent councils may not be welcoming to all income levels or certain subgroups such as Indigenous populations (Alemayehu 2022). In Canadian studies, it was reported that Indigenous populations should also have full control over school food programming and be involved at all stages of development and implementation (Employment and Social Development Canada 2023). School staff were also identified as important partners, suggesting interventions through the school curriculum to develop food literacy (Ismail et al. 2021). However, due to inconsistencies in perceptions from school personnel, adequate communication should be provided to standardize program expectations. Program administrators should also be committed to receiving feedback from participants and their families, and there should be common reporting metrics to effectively evaluate programs (Employment and Social Development Canada 2023).

### Equitable nutrition

Four peer-reviewed articles and one grey literature report (*n* = 5 publications) reported outcomes that show how differences in certain regional and/or sociodemographic factors can influence nutritional needs. In the USA, the student populations that were eligible for the CEP and that were comprised of predominantly African American and Latin American students had lunch selections that met nutrient intake recommendations but were not sufficient in the amounts students consumed. This led the authors to suggest that these school food programs provide more nutrient-dense portions to these groups (Peckham et al. 2019, Adams et al. 2021). Changes in nutrition standards following the implementation of the Healthy, Hunger-Free Kids Act allowed for persisting disparities, because individual schools and districts have autonomy over the foods that are offered within a school food program (Turner et al. 2016, Peckham et al. 2019). While overall positive changes were observed, a study by Turner et al. (2016) found that schools with a majority of Black or Latino students were less likely than a majority of White schools to offer fresh fruit, and schools with low socioeconomic status were less likely compared to schools with students of middle or high socioeconomic status to offer salad on a regular basis. In populations of non-Hispanic Black and White children, participation in the NSLP resulted in higher Healthy Eating Index scores than non-participants (Gearan et al. 2020). This study demonstrated that foods consumed outside of the school lunch period had a negative influence on student diets (with the exception of Hispanic populations), providing insight into the diet quality of certain subgroups that school meals can address. These studies highlight considerations that should be evaluated in accordance with the sociodemographic makeup of schools to ensure fair and equitable access to nutritional meals for students of all backgrounds. Moreover, healthy school meals may mitigate nutritional disparities and lower the prevalence of poor health outcomes in certain equity-deserving groups.

One report from the Canadian literature fell under this theme and provided recommendations from various partners involved in school meal preparation (Employment and Social Development Canada 2023). This included challenges associated with following Canada’s Food Guide due to local nutrition realities, where program administrators may need to adapt school food to account for specific sociodemographic and geographical contexts.

## DISCUSSION

The purpose of this scoping review was to identify considerations that can be applied to increase the diversity, equity, and inclusion of public school food programs. A total of five themes from the review of 21 publications identified key aspects that should be explored further when developing or evaluating a school food program to ensure equitable and fair access to food for all students.

The administration of school food programs played a significant role in the accessibility of school meals and the stigma or lack thereof surrounding food programs. Universal access to school food programs reduced concerns regarding stigmatization and ensured all families were accommodated regardless of their income status, which often predicts eligibility for a program (Cohen et al. 2022). More equitable menu items (e.g. the elimination of higher priced à la carte options), alternative forms of delivery (e.g. grab and go), and care given to serving appealing and tasty food were also deemed important for creating a comfortable and inclusive school food program (Bhatia et al. 2011, Askelson et al. 2017, Datta Gupta et al. 2023). Barriers to program delivery were particularly prominent in rural communities in both Canada and the USA due to transportation and school infrastructure concerns (Employment and Social Development Canada 2023).

The specialized needs and preferences of equity-deserving groups also served as an influencing factor in how school food programs were received. Numerous studies commented on the importance of sociocultural considerations for school foods and there were some correlations found among race/ethnicity, acculturation variables, and participation status (Bailey-Davis et al. 2013, Spruance and Vo 2022). Beyond calls for nutrition equity for minority students, however, there was no mention in the literature about other considerations either for access or the types of food served to indicate what sociocultural consideration would look like for specific ethnic minorities. The Canadian literature held more of a focus on recommendations for Indigenous populations, particularly the importance of integrating traditional teachings to promote awareness of Indigenous cultures (Alemayehu 2022, Employment and Social Development Canada 2023). These conclusions support discussions found in another review where the authors argued that the diversity of cultural food teachings and practices, both in terms of food provided and how it is served, may improve the acceptance of school food programs (Everitt et al. 2023). Additionally, the American Dietetic Association, Society for Nutrition Education, and the American School Food Service Association have stated that school meals are more likely to be selected and eaten when students’ taste and cultural preferences are supported (American Dietetic Association 2003). School food programs that are not culturally appropriate nor inclusive can exacerbate inequities by placing misconceptions about which foods are considered ‘normal’, which can invalidate the experiences of students from diverse backgrounds and disincentivize participation (Freedman 2017). Beyond sociocultural factors, school food programs should also consider how racialized or ethnic minority status in tandem with household income could influence the need for more nutrient-dense foods in programs to mitigate nutritional inequities (Gearan et al. 2020). A perspective not mentioned in the literature is the struggles families face when trying to pack healthy school meals for their children (Hernandez et al. 2018). Due to extenuating circumstances such as mental health issues, poverty, or even a lack of time, parents may be more inclined to provide highly processed foods that are low in nutrients due to convenience. Therefore, students’ overall diets should be considered when planning a menu for a school food program.

Community partner perspectives gathered through qualitative focus groups and interviews served as an important tool when exploring community-specific needs as well as highlighting the need for culturally relevant menu planning (Bailey-Davis et al. 2013, Ohri-Vachaspati 2014). It is important to ensure a sense of agency among partners, particularly parents and students who are currently underrepresented both in program delivery itself and the research literature. Central perspectives gathered throughout the five themes indicated that parents and students are in favour of a universally accessible school food program where meals are prepared and delivered in an appealing manner, cultural preferences are taken into account, and their feedback is sought out and implemented.

It is important to note that these findings and associated discussions are not intended to be generalizable to all communities; while some studies highlight outcomes on nationally representative samples, others report data from sole school districts, regions, states, or provinces. To avoid inaccuracy in representing study results, the terminology used to describe racialized and ethnic groups were directly referenced from the included literature. However, several studies and reports did not include data on specific demographics such as racialized populations and ethnicity, which limits their applicability to other communities. These studies were also limited by their ability to report outcomes on a diverse number of equity-deserving groups, and the use of overarching terminology such as ‘Hispanic’ or ‘Latino’ without considering how members of these allocated groups may prefer to be identified. Therefore, there are challenges presented with applying these themes to specific racialized or ethnic groups due to the generalization of some findings, the lack of representation in some studies (e.g. using ‘Other’ to capture data on Asian and Indigenous populations), and the intersection between factors such as race, ethnicity, and socioeconomic status (Cheng et al. 2015). This highlights gaps in the literature and emphasizes the importance of applying school food considerations to community-specific needs. It is crucial that policymakers, school staff, and program administrators use these themes as a baseline and apply them accordingly to the equity-deserving groups that exist in the populations they are aiming to serve.

In terms of the included publications themselves, there were significantly more peer-reviewed studies in the USA (*n* = 14) compared to Canada (*n* = 4), and all sources were published after 2010. The characteristics of the available literature (years of publication, location) display the recency of this area of research as well as the need for further study, particularly in Canada. The timeframe of included literature (2011–24) can potentially be attributed to the increased recognition of DEI considerations in recent years and the push for transformative change at a societal level. One prominent example of this is the United Nations’ Sustainable Development Goals (SDGs), which were established in 2015 and committed to by both Canada and the USA. A report by Alexander (2021) found that school food programs can contribute to all the SDGs, including reducing inequality within and across countries. It is expected that these frameworks will continue to develop as research moves towards implementing policy with the principles of reconciliation, decolonization, racial justice, and food justice at the forefront (Alexander 2021).

It is also necessary to consider how certain food programs and/or nutrition policy changes may have influenced reported outcomes. For example, the Healthy, Hunger-Free Kids Act passed in 2010 in the USA has undergone several changes since (e.g. increases in portion sizes and regulations on whole grains and sodium), which may alter the results found in studies published after this time (United States Department of Agriculture, 2014; Cohen and Schwartz, 2020).

This research is important given that Canada is in the process of establishing a national school food program but equally crucial for the USA where concerns about DEI and nutritional inequities in school food programs exist (Zhong et al. 2023, Ruetz and Poppendieck 2024). As school food policies develop within Canada, there are numerous learnings that can be adapted from established programs in the USA, including the incorporation of a model similar to the CEP that provides access to all students, steering away from differential (as seen in competitive versus school food offerings) or unappealing food options. The upcoming Canadian school food program should reflect national nutrition standards like the USA but additionally consider the diverse needs and values of communities across the country, which can be achieved through actively seeking feedback from and engaging with participating families.

Given that this review commented on all types of meal programs, future research should further elucidate considerations specific to breakfast, lunch, and snack programs. For instance, SBP participation appears to be lower than NSLP participation due to a variety of factors (Bailey-Davis et al. 2013, Food Research & Action Center 2024). It is important to consider how the aforementioned themes could be applied to each program type and if they vary depending on the equity-deserving group being discussed.

This scoping review held various strengths. The methodology was well-informed by resources such as the PRISMA-ScR as well as Arksey and O’Malley’s framework, which supported the rigour and replicability of the literature search (Arksey and O’Malley 2005, Tricco et al. 2018). The additional consultation of the literature validated the thematic analysis framework used in this review (Braun and Clarke, 2006; Clarke and Braun, 2017). This resulted in a thorough search of peer-reviewed publications and grey literature sources. From the authors’ knowledge, this is one of the first scoping reviews focused on DEI practices in school food programs, which supplements and comments on the state of current literature available. In terms of limitations, there are some equity-deserving groups and intersectional identities of students that were potentially not captured by the search (e.g. religious affiliations). The application of these results may differ depending on these factors that were not discussed. The methodology of a scoping review itself, moreover, limits the ability to critically appraise the quality of literature included and pool results; thus, the quality of evidence was not formally reviewed and numerical data analysis could not be conducted on quantitative results (Tricco et al. 2018). Although thematic analysis was carried out to adhere to all study types, supplementing this interpretative approach with statistical relationships could have better elucidated causal patterns within the included studies.

## CONCLUSION

Government-funded school food programs have the potential to serve as an essential resource for the nutritional health of school-aged children and can cultivate a positive food environment within schools. However, a lack of consideration for underrepresented and/or equity-deserving groups limits their impact leading to underutilization alongside social and nutritional inequities. This review went beyond the consideration of equity-deserving groups to acknowledge socioeconomic status and urban/rural school locations, which play a role in equity and fairness in access to nutritious school food programs. The themes highlighted in this review (universal access, food preparation and delivery, sociocultural food preferences, partner involvement, and equitable nutrition) should be used as a starting point to inform future research in specific equity-deserving populations, high-poverty schools, school food program types, and grade levels. These developments are essential for accommodating diverse groups of students with varied needs through school food programs.

## Data Availability

The data underlying this research is available within the materials available in this article.
